# Prediction of neurocritical care intensity through automated infrared pupillometry and transcranial doppler in blunt traumatic brain injury: the NOPE study

**DOI:** 10.1007/s00068-023-02435-1

**Published:** 2024-01-16

**Authors:** Pierluigi Banco, Fabio Silvio Taccone, Dimitri Sourd, Claudio Privitera, Jean-Luc Bosson, Thomas Luz Teixeira, Anais Adolle, Jean-François Payen, Pierre Bouzat, Tobias Gauss

**Affiliations:** 1 Department of Anaesthesia and Intensive Care, Univ. Grenoble Alpes, Centre Hospitalier Universitaire Grenoble, and Inserm, U1216, Grenoble Institut Neurosciences, 38000 Grenoble, France; 2 Department of Intensive Care, Hôpital Universitaire de Bruxelles (HUB), Université Libre de Bruxelles (ULB), Brussels, Belgium; 3 Department of Public Health, Univ. Grenoble Alpes, Centre Hospitalier Universitaire Grenoble Alpes, Grenoble, France; 4 School of Optometry and Vision Science, University of California, Berkeley, Berkeley, CA USA

**Keywords:** Traumatic brain injury, Neurocritical care, Transcranial doppler, Pupillometry

## Abstract

**Purpose:**

This pilot study aimed to determine the capacity of automated infrared pupillometry (AIP) alone and in combination with transcranial doppler (TCD) on admission to rule out need for intense neuroAQ2 critical care (INCC) in severe traumatic brain injury (TBI).

**Methods:**

In this observational pilot study clinicians performed AIP and TCD measurements on admission in blunt TBI patients with a Glasgow Coma Score (GCS) < 9 and/or motor score < 6. A Neurological Pupil index (NPi) < 3, Pulsatility Index (PI) > 1,4 or diastolic blood flow velocity (dV) of < 20 cm/s were used to rule out the need for INCC (exceeding the tier 0 Seattle Consensus Conference). The primary outcome was the negative likelihood ratio (nLR) of NPi < 3 alone or in combination with TCD to detect need for INCC.

**Results:**

A total of 69 TBI patients were included from May 2019 to September 2020. Of those, 52/69 (75%) median age was 45 [28–67], median prehospital GCS of 7 [5–8], median Injury Severity Scale of 13.0 [6.5–25.5], median Marshall Score of 4 [3–5], the median Glasgow Outcome Scale at discharge was 3 [1–5]. NPi < 3 was an independent predictor of INCC. NPi demonstrated a nLR of 0,6 (95%CI 0.4–0.9; AUROC, 0.65, 95% CI 0.51–0.79), a combination of NPi and TCD showed a nLR of 0.6 (95% CI 0.4–1.0; AUROC 0.67 95% CI 0.52–0.83) to predict INCC.

**Conclusion:**

This pilot study suggests a possible useful contribution of NPi to determine the need for INCC in severe blunt TBI patients on admission.

## Introduction

The epidemiology and management of traumatic brain injury (TBI) has evolved considerably over the last decade [1]. The initial assessment of TBI patients is crucial in predicting the likelihood of subsequent neuro-worsening. Together with the Glasgow Coma Scale (GCS) and the cerebral CT-scan performed on admission, pupil examination is a cornerstone of this assessment [2], but manual examination lacks precision and reliability [3, 4]. Automated infrared pupillometry (AIP) offers a reliable alternative, and the Neurological Pupil Index (NPi) integrates several parameters of pupillary reactivity into one algorithm [5, 6]; an NPi value below 3 indicates impaired pupillary reactivity and has been shown to predict intracranial pressure and neurological complications, providing prognostic information before anisocoria or mydriasis develop [7–11]. A recent prospective multicenter study confirmed the prognostic value of serial NPi measurements [12]. Sequential NPi measurements can be considered an early indication for osmotherapy and decompressive surgery [13, 14] in these patients. Whether admission NPi assessment is useful in this setting remains poorly described.

In a recent retrospective study including 100 TBI patients, NPi on admission showed a moderate accuracy to predict unfavorable neurological outcome; no data on the prediction of the intensity of care was provided [15]. Transcranial Doppler (TCD) could be a relevant non-invasive tool to help identifying patients at risk of increased intracranial pressure (ICP) after head trauma [16]. However, while abnormal TCD findings could predict neuro-worsening in mild to moderate TBI patients [17] few data on the role of early TCD assessment on the prediction of intensity of care after TBI are available. In one study [16] all TBI patients with abnormal TCD findings on admission further required osmotherapy and/or norepinephrine.

As such, it appeared justified to assess the usefulness of NPi measurements on admission to rule out the need for intensive neurocritical care (INCC), alone and in combination with TCD. In this study, we therefore tested the hypothesis that early abnormalities in NPi and TCD findings has a sufficient negative predictive capacity to rule out the need for intense neurocritical care (INCC) in TBI patients.

## Methods

### Study design

This was an observational pilot study carried out in the Resuscitation Rooms and Intensive Care Units of the Hôpital Universitaire de Bruxelles (HUB), Brussels, and the University Hospital of Grenoble, Grenoble, France. The study received approval from both institutional ethics committees. AIP and TCD were part of routine monitoring on admission for patients with TBI, and the results were recorded into the patient data monitoring system. Inclusion and data collection were conducted between May 2019 and August 2020, and data analysis took place between November 2022 and February 2023. Follow-up was performed for the duration of the patients' stay in the Intensive Care Unit. The study adhered to the STROBE checklist. The NOPE study was authorized by the Direction of Research and Innovation from the Grenoble Alpes University Hospital in according to French law as observational, retrospective study (MR004, authorization 12/11/2020, DRCI CHUGA). The study was registered with the French Agency for Data and Privacy Protection (CNIL, declaration 2,205,066 v 0). All participating patients obtained written information to be able to withdraw their participation. The study was conducted in agreement with the Code of Ethics of the World Medical Association (Declaration of Helsinki).

### Study population

All patients admitted to both centers with suspicion of blunt TBI were considered for inclusion. The inclusion criteria were a pre-hospital Glasgow Coma Scale (GCS) score of less than 9 and/or a GCS motor scale score of less than 6 upon hospital admission, and the presence of visible intracranial lesions on admission cerebral CT-scan. Patients who were expected to die imminently were excluded from the study. Trained physicians conducted Transcranial Doppler (TCD) on admission. Management of TBI patients were performed according to the Seattle Consensus Conference [18, 19].

### Data collection

The study collected demographic data, comorbidities, and severity scores including the Simplified Acute Physiology Score (SAPS)-2 score [20], Glasgow Coma Scale [21], Abbreviated Injury Scale (AIS) and Injury Severity Scale (ISS) [22] of all patients. The mechanism of trauma, such as road traffic accidents, falls, and other factors, clinical pupil examination, and admission CT-scan findings were also recorded. The study also documented all therapies administered to reduce ICP, including the therapy intensity level (TIL) recorded for both TILBasic and TILSum [23]. Information on the length of stay in the ICU, duration of mechanical ventilation, length of hospital stay, mortality at day 14 and the extended Glasgow Outcome Scale (GOS) at hospital discharge were also collected. An unfavorable neurological outcome corresponded to extended Glasgow Outcome Scale of 1–2 (GOSe).

### Measurements

In this study, the NPi-200 pupillometer (Neuroptics, Irvine, CA, USA) was utilized. This device uses an infrared camera that integrates a calibrated light stimulation of fixed intensity (1000 Lux) and duration (3.2 s) to measure the pupil size and dynamic pupillary variables (including percentage constriction, latency, constriction velocity, and dilation velocity) with a limit of 0.05 mm. The NPi, based on the integration of these variables into an algorithm, is directly provided, resulting in a scalar index with values ranging from 0 to 5, with a decimal precision of 0.1. Pathological NPi values were defined as less than 3.0, consistent with previous reports [7]. Trained nurses or physicians collected NPi from both eyes on admission.

TCD was also performed by trained physicians using the temporal window on both sides and an echo-color Doppler device with a 2-MHz transducer; measurements were performed bilaterally on the middle cerebral artery (MCA); dV and PI [(systolic velocity – diastolic velocity)/mean velocity] were recorded. A PI > 1.2, > 1.4 and dV < 20 cm/s were considered abnormal [16, 17].

### Study outcomes

The primary outcome was the negative likelihood ratio of NPi alone and in combination with TCD measurements to rule out the need of Intensive Neuro Critical Care (INCC). INCC was defined as interventions exceeding the tier 0 of the Seattle Consensus Conference for TBI management [18, 19]. Secondary outcomes included the positive likelihood ratio, sensitivity, specificity, prevalence, positive and negative predictive value, receiver operating curves characteristics to express the capacity to diagnose the need of Intensive Neuro Critical Care (INCC), unfavorable neurological outcome at hospital discharge (i.e. eGOS 1–4) and 14-day mortality.

### Statistical analysis

A statistician from the Grenoble Alpes Biometrical Department performed all the analyses. Descriptive analysis was carried out using the mean and standard deviation or median and interquartile ranges, as appropriate. The normal distribution was assessed by graphical estimation. Mann-Whitney test and Chi-square test were used to analyze continuous and categorical parameters, respectively the alpha level of 0.05 was set for all analyses. The association between NPi, PI, dV, age, Simplified Acute Physiology Score (SAPS)-2, Glasgow Coma Scale (GCS) motor score, in-hospital mortality, TILbasic and TILmax and Glasgow Outcome Scale extended (GOSe) was explored using two-by-two Pearson correlation coefficients for continuous variables and correlation for categorical variables. A linear multiple, backward regression tested the independent association of NPi to all Tier levels 0–3. The model included the following variables, in addition to NPi < 3: age, SAPS-2, PI > 1.2, > 1.4 and diastolic velocity 20 m/s (dV), Glasgow Coma Motor Scale and AIS head > 3 (Abbreviated Injury Scale). Diagnostic thresholds were determined using the Youden index method. A probability threshold of 0.05 was set to accept or reject variables to retain in the model, and the squared predictive quadratic error determined. No specific treatment was applied to missing data. All analyses were performed using (Stata 18, 4905 Lakeway Drive, College Station, Texas USA).

## Results

### Study population

**Fig. 1:**
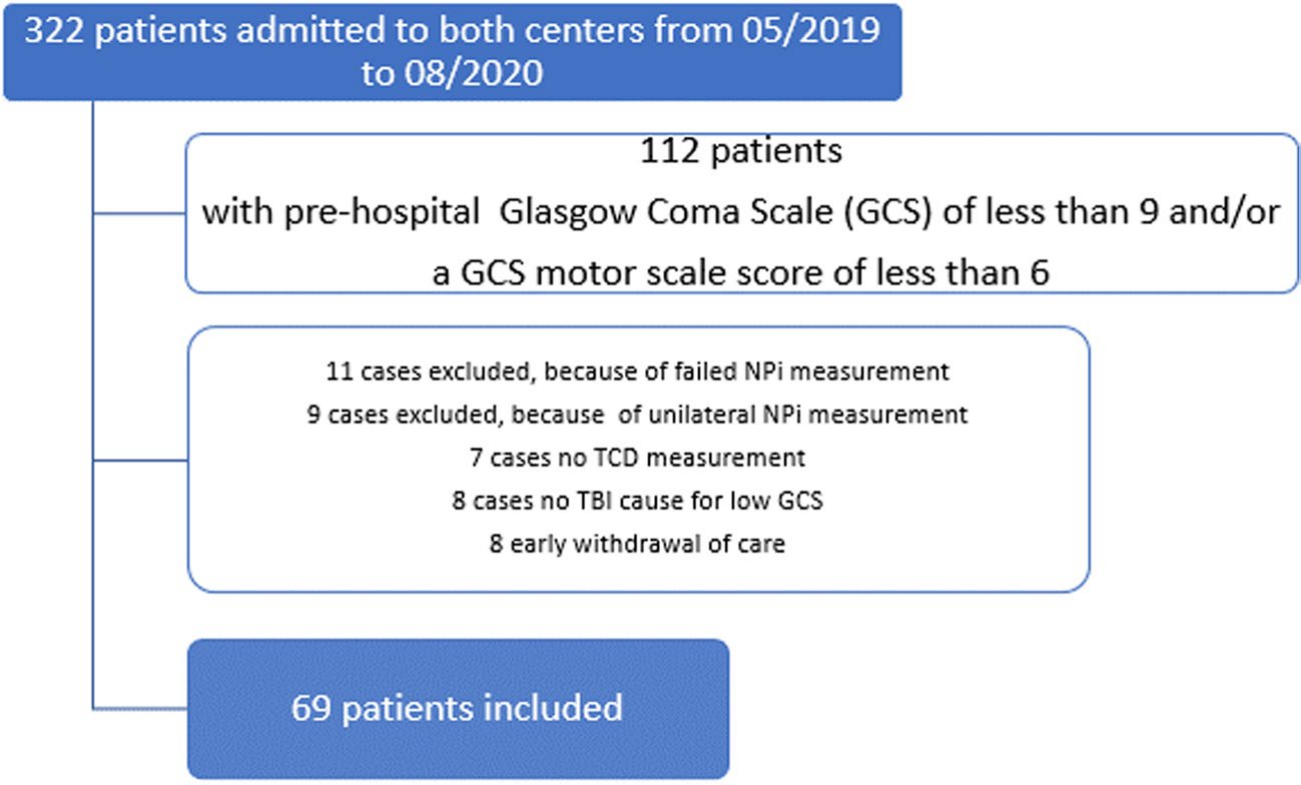
Flowchart of study

Over the study period, investigators screened 322 patients with suspicion of TBI admitted to both participating centers; 112 patients corresponded to the inclusion criterion and 69 were included (Fig. 1). Of those, 52/69 (75%) patients were male, Median age was 45 [28–67] years, median prehospital GCS was 7 [5–8], median Injury Severity Scale was 13 [6.5–25.5] and median AIS head was 4 [3–5], median GOSe at hospital discharge [1–5]. A total of 22/69 (31%) patients died before hospital discharge or had unfavourable neurological outcome (GOSe 1–2). Table 1 lists the characteristics of the included patients.

**Table 1:** Characteristics of the study population

	*N* = 69
Age, years	45 [28–67]
Male gender, *n* (%)	52/69 (75%)
Trauma mechanism, *n* (%)	
Road Accident	18/69 (26%)
Fall	45/69 (65%)
Other	6/69 (9%)
Prehospital GCS	7 [5–8]
GCS motor score	4 [3–5]
Prehospital abnormal pupils, *n* (%)	34/69 (49%)
Prehospital Osmotherapy, *n* (%)	19/69 (28%)
Prehospital Intubation, *n* (%)	49 (71%)
Marshall Score on CT-scan	4 [3–5]
Rotterdam Score on CT-scan	4 [3–5]
Crash Score on day 6	76.5 [51.2–92.2]
Crash Score on day 14	35.6 [11.5–71.8]
Injury Severity Scale	13 [6.5–25.5]
AIS median head	4 [3–5]
AIS Head > 3	48/69 (69%)
AIS median other than head	2 [1–3]
SAPS 2 score	40.2 (15.6)
Catecholamine use	52/69 (75%)
Extracranial surgery	19/69 (27%)
External ventricular drain	16/169 (23%)
Neurosurgery	22/69 (31%)
Decompressive Craniectomy	10/69 (14%)
Any episode of increased intracranial pressure (> 20 mmHg)	37/69 (55%)
NPi on admission, median [IQR]	3.3 [1.8–4.1]
NPi < 3	30/69 (43%)
Pulsatility index (PI) on admission, mean (SD)	1.11 (0.37)
Diastolic velocity (dV) on admission, cm/s, mean (SD)	35/69 (17.01)
Abnormal transcranial doppler (TCD) (PI > 1.4 or dV < 20)	31/69 (44%)
Maximum tier of ICP therapy	2 [1–3]
Crash Score on day 6	76.5 [51.2–92.2]
Crash Score on day 14	35.6 [11.5–71.8]
ICU length of stay, days	12 [3–23]
Intrahospital Mortality, n (%)	22/69 (31%)
Glasgow Outcome extended Score (GOSe), median [IQR]	3 [1–5]
Unfavorable Neurological Outcome, GOSe 1–2, *n* (%)	22/69 (31%)

Data are reported a mean (SD), median (IQR) or absolute count (%)

### NPi and TCD assessment on admission

**Fig. 2:**
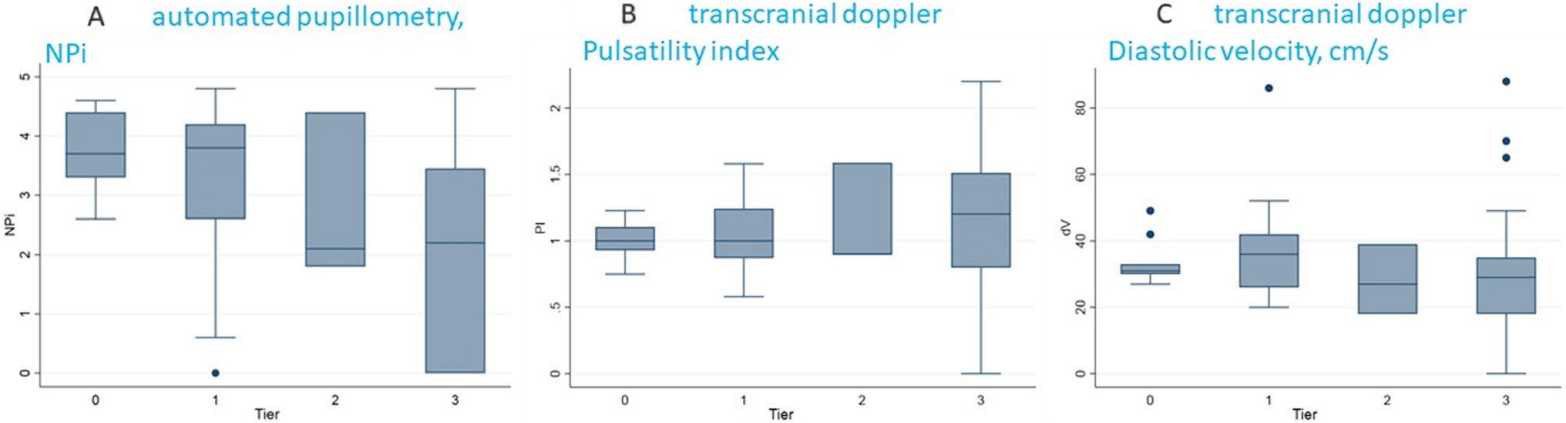
Distribution of automated pupillometry, NPi, and transcranial doppler, pulsatility index (PI) and diastolic velocity (dV, cm/s), values according to intensity of neurocritical care (INCC) tiers 0–3; panel **A**, automated pupillometry, NPi; panel **B**, transcranial doppler, pulsatility index; panel **C**, transcranial doppler, diastolic velocity, cm/s

Median NPi on admission was 3.3 [1.8–4.1], 39% (27/69) patients had an NPi < 3. Mean PI was 1,03 (SD 0,35), mean dV 35 cm/s (SD 17); 44% (31/69) had an abnormal PI (> 1.2 or > 1.4) and/or dV (< 20 cm/s). Figure 1 illustrates the distribution of NPi and PI and diastolic velocity values across different INCC levels (tiers 0–3) (Fig. 2).

### NPi, TCD and INCC

Median tier of neurocritical care was 2 [1;3]; 61/69 (88%) patients received INCC (> tier 0) and 33/69 (47%) received care > tier 3. Patients receiving INCC had lower NPi or dV and higher PI values than others (Fig. 1).

The multiple linear regression identified the NPi < 3 as independent predictor of any tier 0–3. The final model retained NPi < 3 and SAPS-2.

**Fig. 3:**
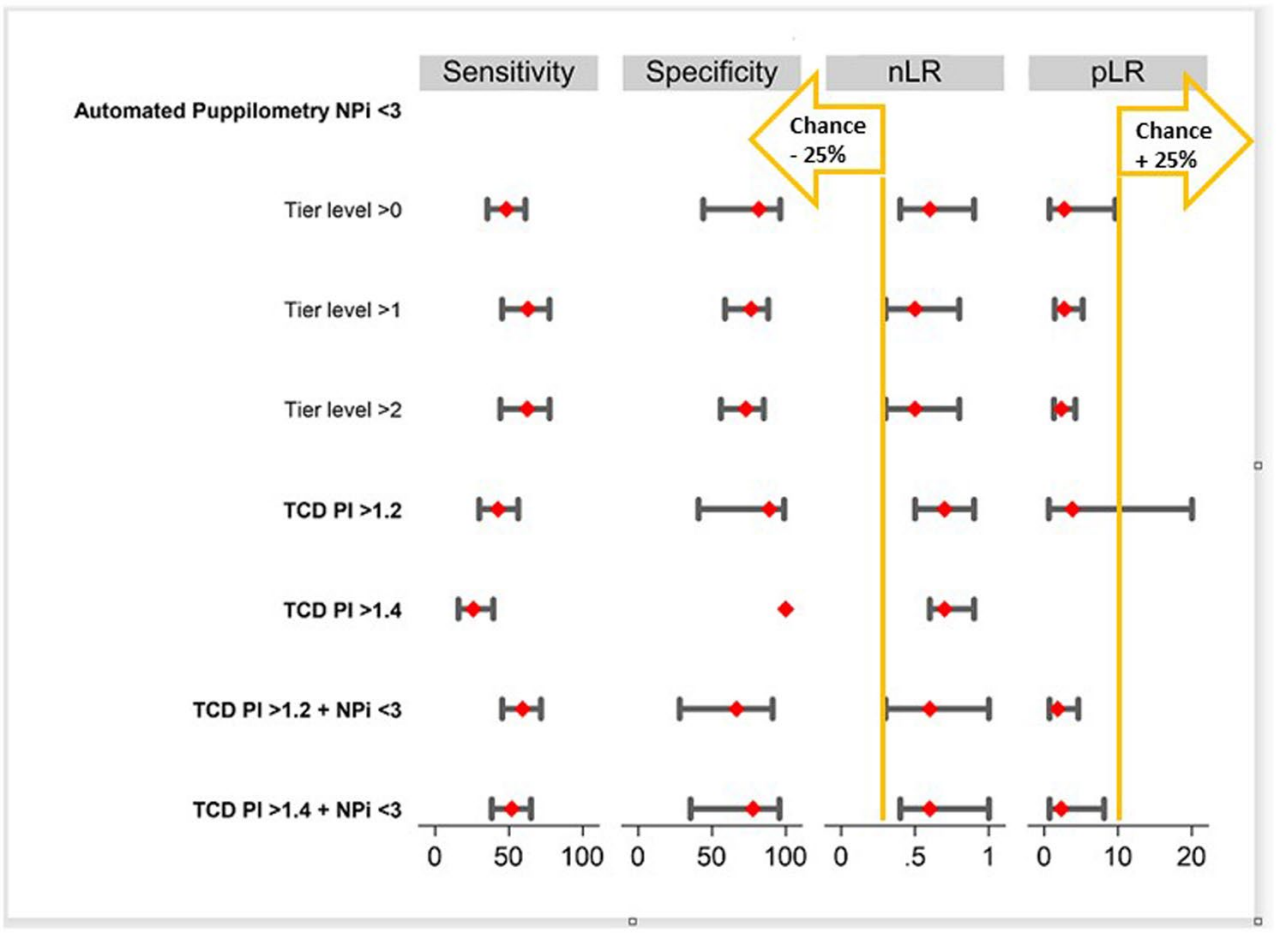
Diagnostic performance of NPi < 3, Transcranial Doppler PI > 1,2 and 1.4 to detect need for intensive neurocritical care (INCC); negative Likelihood ratio < 0,3 reduces chance of need of INCC by 25%, positive Likelihood ratio > 10 increases chance of need for INCC by 45%

Table 2 summarizes the results of the diagnostic performance of NPi and TCD for different tier levels. In brief, NPi < 3 alone and TCD > 1.2 or 1.4 for a combination of NPi and TCD show comparable diagnostic performance to detect patients with INCC with positive likelihood ratios ranging from 1.8 to 3.8 and negative likelihood ratios from 0.5 to 0.7. The primary outcome negative likelihood ratio of NPi < 3 to detect need for INCC (tier > 0) was 0. For recall, a negative likelihood ratio < 0.3 reduces the chance of a need of INCC by 25%, a positive likelihood ratio > 10 increases the chance of a need for INCC by 45%. Figure 3 recapitulates sensitivity, specificity, positive and negative likelihood ratios for different tier levels and different thresholds.

**Table 2:** Summary diagnostic performance of the different diagnostic modalities NPi < 3, PI > 1,2 and PI > 1,4

	Sensitivity	Specificity	Prevalence	PPV	NPV	pLR	nLR
NPi < 3 to detect need for INCC							
Tier > 0	48.3% [35.5–61.2]	81.8% [44.1–96.3]	84.1% [73.2–91.0]	93% [76–98]	23% [12–39]	2.7 [0.7–9.6]	0.6 [0.4–0.9]
Tier > 1	62.9% [45.4–77.5]	76.5% [58.8–88.1]	50% [39–62]	73% [54–86]	67% [50–80]	2.7 [1.4–5.2]	0.5 [0.3–0.8]
Tier > 2	62.5% [44.2–77.8]	73.0% [56.0–85.1]	46.4% [34.8–58.3]	67% [48–82]	69% [53–82]	2.3 [1.3–4.2]	0.5 [0.3–0.8]
Transcranial Doppler PI > 1.2 to detect need for INCC							
Tier > 0	42.6% [29.9–56.3]	88.9% [40.9–98.9]	84.1% [73.2–91.0]	95.8% [73.5–99.5]	20.5% [10.4–36]	3.8 [0.6–25.0]	0.7 [0.5–0.9]
Transcranial Doppler PI > 1.4 to detect need for INCC							
Tier > 0	25.9% [15.8–39.5]	100	84.1% [73.2–91.0]	100	18.4% [9.7–32.1]	NA	0.7 [0.6–0.9]
Combination of NPi < 3 AND Transcranial Doppler PI > 1.2 to detect need for INCC							
Tier > 0	59.3% [45.5–71.7]	66.7% [28.1–91.1]	84.1% [73.2–91.0]	91.4% [75.8–97.3]	21.4% [9.6–41.2]	1.8 [0.7–4.6]	0.6 [0.3–1.0]
Combination of NPi < 3 AND Transcranial Doppler PI > 1.2 to detect need for INCC							
Tier > 0	51.9% [38.4–65.0]	77.8% [35.5–95.7]	84.1% [73.2–91.0]	93.3% [75.8–98.4]	21.2% [10.2–39]	2.3 [0.7–8.1]	0.6 [0.4–1.0]

All values with 95% CI; *INCC* intensity of neurocritical care, *PI* pulsatility index, *PPV* positive predictive value, *NPV* negative predictive value, *pLR* positive likelihood ratio, *nLR* negative likelihood ratio, *INCC* intensity of neurocritical care

**Fig. 4:**
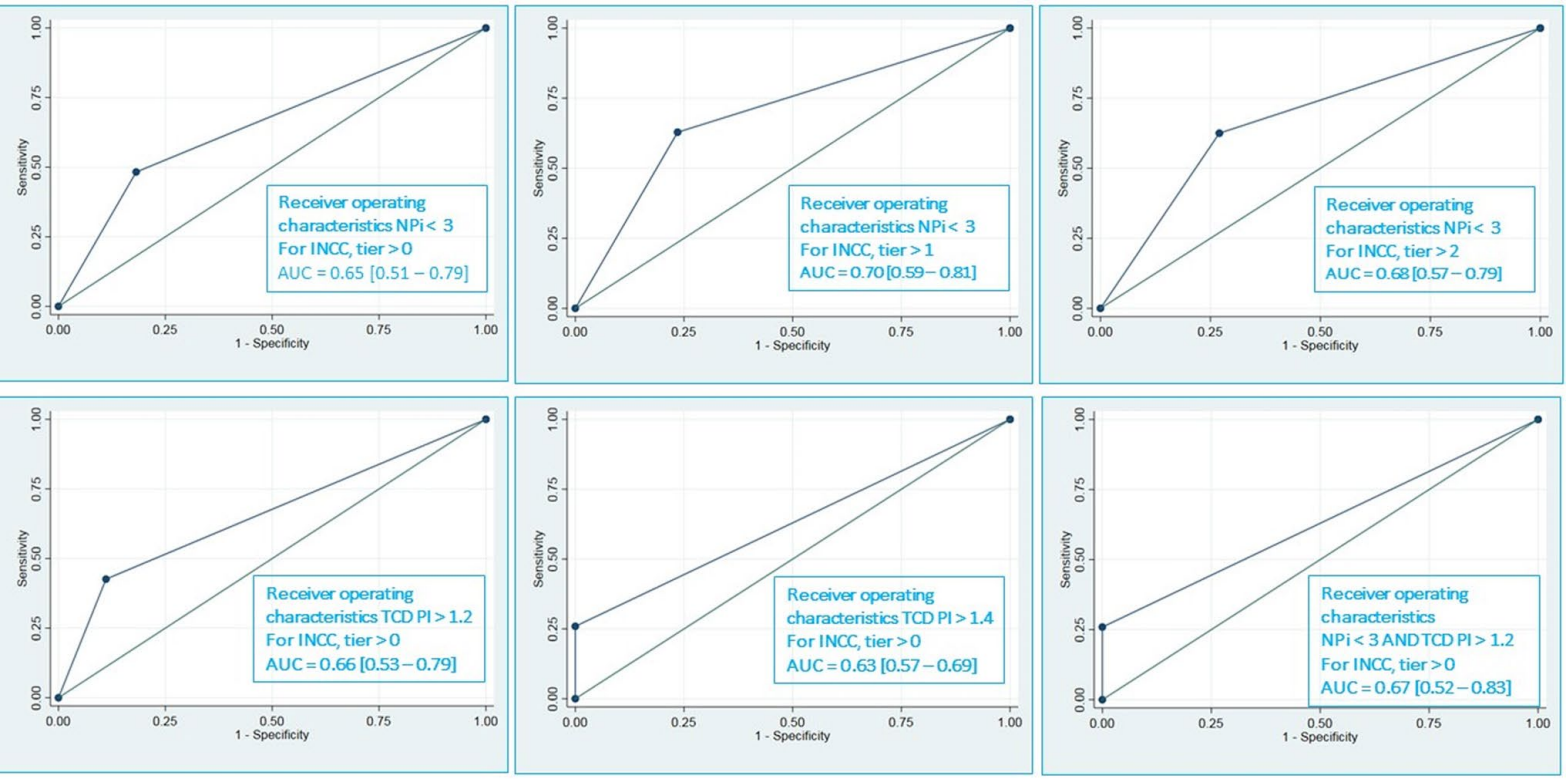
Receiver operating characteristics for panel A-F: A) NPi < 3 for INCC, tier > 0; B) NPi < 3 for INCC, tier > 1; C) NPi < 3 for INCC, tier > 2; D) TCD PI > 1.2 for INCC, tier > 0; E) TCD PI > 1.4 for INCC, tier > 0; F) NPi < 3 AND TCD PI > 1.2 for INCC, tier > 0

The Odds Ratio for a decreased NPi and increase mortality was 1.93 [95% CI 1.34–2.79, *p* = 0.000] with an ideal threshold for the NPi of 1.95 according to the method of Youden. The Odds Ratio for decreased neurological outcome for decreasing NPi was 1.53 [95% CI 1.08; 2.2, *p* = 0.016] with an ideal threshold for the NPi of 3.55. Figure 4 plots the receiver operating characteristics of NPi and TCD for different tier levels to detect a need for INCC. The AUC ranges from 0,63 [95% CI 0.57–0.69] for TCD PI > 1.4 to 0.7 to predict INCC > tier 0 [95% CI 0.59–0.81] for NPi < 3 to predict INCC > tier 1.

The NPi was significantly associated with intrahospital mortality (Pearson correlation coefficient, 0.6172, *p* = 0.05), and with GOSe at hospital discharge (Pearson correlation coefficient 0.4937, *p* = 0.0001). A multivariable regression including NPi < 3, pulsatility index (PI) > 1,4 and diastolic velocity (dV) < 20 cm/s does not demonstrate an association with intrahospital mortality and GOSe neither with NPi < 3, nor PI. The association appears only with dV.

## Discussion

This retrospective two-center pilot study suggests the potential usefulness of a pupillometric measurement on arrival in the resuscitation room to rule out the need for INCC in blunt traumatic brain injury. The pupillometric composite measure, NPi performs comparably to the TCD to identify the need for neurocritical care. The combination of NPi and TCD in the resuscitation room may provide a useful indication to determine the need for intense neurocritical care.

The recent multicenter ORANGE study confirmed the reliability and validity of serial NPi measurement for prognostic information in acute brain injury including TBI [12]. Several studies assessed the triage capacity of automated pupillometry in patients with TBI at different stages in their clinical course. Jahns et al. observed in a sample of 54 patients within the first 24 h of admission to the ICU that patients with sustained episodes of raised intracranial pressure (ICP) had consistent decrease of NPi [13]; patients with a low NPi and raised ICP showed worse neurological outcome at six months. Trent et al. used automated pupillometry as triage tool in a prospective study to identify patients within the first 24 h for risk of neurological deterioration (drop of 2 GCS points) in a sample of 95 TBI patients [24]. Their observation provided a sensitivity of 51.4% and specificity of 91.7%. Park et al. observed a correlation between low GCS and low NPi. El Ahmadieh et al. documented a correlation of NPi < 3 and raised ICP and need for neurosurgery [14].

In contrast, Stevens et al. found a weak relationship between initial NPi measurement and subsequent episodes of raised ICP in a sample of 40 TBI patients [25]. Furthermore, Teixeira et al. performed the most advanced study on pupillometry in TBI patients [15]. Their sample is very comparable to the present study, a homogenous group of 100 severe TBI patients with a median Marshall score of 5 and NPi measurement performed rapidly after admission. The investigators observed that lower NPi are associated with worse outcome and raised ICP and higher level of care (TILSUM score) but question the predictive value of NPi for medium term outcomes such as GOSe on discharge from the ICU.

The present study differs to previous studies in so far as the measurement was performed in the resuscitation room on admission and with the specific goal to predict the level of neurocritical care. The investigators felt this prediction to be more useful to clinicians in the initial phase than mortality or medium and long- term outcome. Prediction of raised ICP and neurocritical care level remains challenging even in expert centers with the currently available tools [3]. Underestimation of the required neurocritical care can lead to insufficient neuroprotection and premature wake up trials with deleterious effects on cerebral hemodynamics and compliance. Stratification of this risk and patient needs with an objective measurement such as NPi would be a helpful addition to the diagnostic arsenal.

All established prognostic TBI scores (Crash, IMPACT) integrate information on pupil anomalies as a binary variable, present or not present only retain the pupil status based on human observation. None of the existing scores integrates quantitative information derived from automated pupillometry. Pupil changes in TBI can be too subtle to be captured by the human eye [4]. An automated measurement increases the capacity to detect subtle changes. Furthermore, causes for pupil changes after TBI are heterogenous and not exclusively a consequence of brain herniation. Pupil changes can occur after direct ocular or optic nerve trauma, injury of the pathway of pupillometric fibers or coordinating nuclei and pathways in the brainstem. Integration of quantified automated pupil anomalies into the established scores might improve their predictive performance and capacity to individualize prediction and care. As shown in this study, used as standalone modality, NPi < 3 offers acceptable negative predictive value and negative likelihood ratios [3, 4] to indicate a low probability to require intense levels of neurocritical care. Compared to other modalities, automated pupillometry is non-invasive, simple, and rapid to use without a particular training or skill and easy to repeat to allow serial assessment.

The present study is the first to suggest a diagnostic synergy of TCD and NPi to rule out the need for INCC. This finding offers avenues for new research and development of predictive scores that include the information of automated pupillometry and TCD. The existing scores were established from cohorts before 2008. TBI epidemiology and management have evolved considerably since then, an update might be indicated.

## Limitations

This pilot study is a small sample admitted to two centers. A selection bias cannot be excluded. The investigators did not perform serial measurements to document NPi and TCD changes over time. Some TCD measurement are missing, because no signal could be obtained, or because the measurement was not performed for various reasons (patient in shock, low perfusion pressure). Despite measurements being performed by trained team members and not independent operators, the context of the resuscitation room may not provide measurement conditions equal to a laboratory in particular regarding ambient light conditions.

## Conclusion

This pilot study suggests some usefulness of a single automated pupillometry to rule out the need for INCC in blunt TBI patients on admission. The diagnostic performance of NPi seems comparable to a single transcranial doppler measurement. Their combination to identify patients in need for neurocritical care could be synergistic. These preliminary results open new avenues of research for the development of TBI prediction scores integrating pupillometry and transcranial doppler measurements.

## Data Availability

The complete study data set is available upon request.

## Abbreviations

*AIP*: Automated infrared pupillometry
*INCC*: Intense neuro critical care
*TBI*: Traumatic train Injury
*TCD*: Transcranial Doppler
*NPi*: Neurological Pupil index
*PI*: Pulsatility Index
*nLR*: Negative likelihood ratio
*AUROC*: Area under receiver operating curve
*MCA*: Middle cerebral artery
*dV*: Diastolic velocity
*GOSe*: Glasgow Outcome Scale extended
